# Acute myeloid leukemia with *IDH1* and *IDH2* mutations: 2021 treatment algorithm

**DOI:** 10.1038/s41408-021-00497-1

**Published:** 2021-06-03

**Authors:** Ghayas C. Issa, Courtney D. DiNardo

**Affiliations:** Department of Leukemia, The University of Texas MD Anderson Cancer Center, Houston, TX USA

**Keywords:** Cancer genetics, Drug development, Cancer therapy

## Abstract

Acute myeloid leukemia is a genetically heterogeneous hematologic malignancy; approximately 20% of AML harbors a mutation in the isocitrate dehydrogenase (*IDH*) genes, *IDH1* or *IDH2*. These recurrent mutations in key metabolic enzymes lead to the production of the oncometabolite 2-hydroxyglutarate, which promotes leukemogenesis through a block in normal myeloid differentiation. Since this discovery, selective oral inhibitors of mutant IDH1 and IDH2 have subsequently been developed and are now approved as single agent therapy, based on clinical efficacy observed within the original first-in-human trials. The investigation of IDH inhibitors in combination with standard therapies such as azacytidine, with intensive chemotherapy, and with other small molecule targeted therapies in rational combinations are currently under evaluation to further improve upon clinical efficacy.

## Introduction

In concert with the sequencing of the first AML genome in 2008, the discovery of isocitrate dehydrogenase (IDH) mutations in AML was first described^1^. It is now recognized that approximately 8% and 12% of acute myeloid leukemias harbor an *IDH1* or *IDH2* mutation, respectively, with IDH mutations also present in a minority of other myeloid malignancies such as myelodysplastic syndrome (MDS) and accelerated myeloproliferative neoplasms (MPNs)^2,3^.

Mutations in *IDH1* and *IDH2* occur at conserved arginine residues within the enzymatic active site, specifically the R132 locus of IDH1, and the R140 or (less commonly) the R172 locus of IDH2^4^. These cancer-associated mutations cause a loss-of-function in the normal Kreb’s cycle reaction of isocitrate to alpha-ketoglutarate (aKG), and instead trigger a reverse reaction where aKG is reduced to 2-hydroxyglutarate (2-HG)^5,6^. In this manner, 2HG functions as a cancer “oncometabolite”, competitively inhibiting aKG-dependent enzymes such as the TET family enzymes and lysine demethylases, and leading to a characteristic hypermethylated phenotype^7^. The pathophysiology stemming from aberrant 2HG production also occurs through abnormal H1F1a and induction of BCL2 dependence via inhibition of cytochrome C oxidase^8,9^.

The impact of *IDH* mutations on AML prognosis remains somewhat controversial, although a generally inferior outcome is seen with *IDH1* mutations and a relatively favorable prognosis may be seen with *IDH2* mutations, particularly R172K IDH2 mutations, in the setting of standard intensive chemotherapy^10–17^. *IDH1* and *IDH2* mutations are associated with certain patient and disease-specific characteristics; notably an older age at presentation, diploid or other intermediate-risk cytogenetics (i.e. trisomy 8), and a sustained platelet count at presentation. The impact of *IDH1* and *IDH2* mutations are also context dependent, with *IDH* mutations that occur in the setting of *NPM1* mutations (without *FLT3*-ITD) appearing to confer more favorable outcomes^12^.

**Fig. 1:**
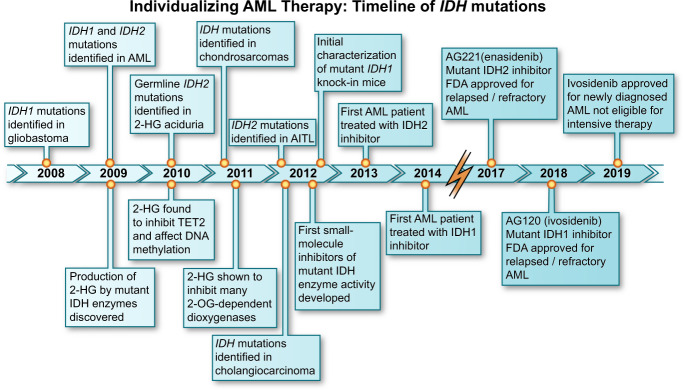
A timeline depicting the decade of progress in *IDH*-mutated malignancies. IDH, Isocitrate Dehydrogenase; 2HG, 2-hydroxyglutarate; AML, acute myeloid leukemia; TET2, Ten-Eleven Translocation-2; 2-OG, 2-oxygluterate; AITL, angioimmunoblastic T-cell lymphoma.

The recent discovery of recurrent *IDH* mutations in cancer led to the rapid development of small molecule targeted inhibitors, which effectively inhibit the 2HG oncometabolite and promote the restoration of normal myeloid differentiation^18^. In less than 10 years from the original discovery of cancer-associated *IDH1* and *IDH2* mutations, first-in-class mutant IDH1 and IDH2 are now clinically available for patients with AML harboring these mutations. (Fig. 1)

**Fig. 2:**
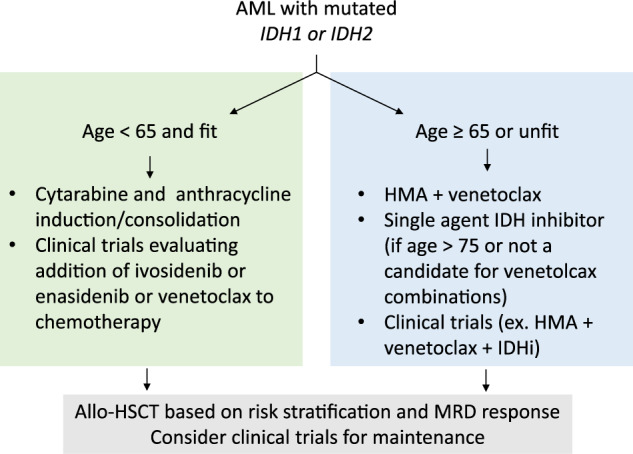
Treatment algorithm for AML with mutated *IDH1* or *IDH2*. AML, acute myeloid leukemia; *IDH*, *Isocitrate Dehydrogenase*; HMA, hypomethylating agent; IDHi, IDH inhibitor; Allo-HSCT, allogeneic hematopoietic stem cell transplant; MRD, minimal or measurable residual disease.

This review will focus on current strategies of IDH1 and IDH2 inhibition in AML (Fig. 2). Table 1 provides a summary of the reported trials and efficacy outcomes seen with IDH1 and IDH2 inhibitors to date.

**Table 1:** Clinical activity of IDH inhibitors as single agents or in combination.

A	7 + 3	V	E	I	N	Md age	CR/CRi	mOS	References
**Newly Diagnosed AML:**									
A					28	78 yrs	10.7%	6.2 mo	^41,42^
A					33	75 yrs	24.2%	22 mo	^24^
			E		39	77 yrs	21%	11.3 mo	^23^
				I	33	76 yrs	48.5%	12.6 mo	^29^
A		V			79	76 yrs	78.5% (85.7% *IDH2*, 65.6% *IDH1*)	24.5 mo (NR for *IDH2*, 17.5 mo *IDH1*)	^42^
A			E		68	75 yrs	63.2%	22 mo	^24^
A				I	23	76 yrs	69.6%	NR (12 mo OS 82%)	^30^
A		V		I	12	67 yrs	75%	NR	^43^
	7 + 3		E		91	63 yrs	74% (80% de novo, 63% sAML)	12-mo OS 77%	^25^
	7 + 3			I	60	63 yrs	77% (88% de novo, 50% in sAML)	12-mo OS 78%	^25^
**Relapsed/Refractory AML:**									
			E		345	68 yrs	28.6%	9.3 mo	^20^
				I	258	68 yrs	30%	8.8 mo	^27^

*A* azacytidine, *V* venetoclax, *E* Enasidenib, *I* Ivosidenib, *Md age* median age, *yrs* years, *mo* months, *CR/CRi* complete response/complete response with incomplete count recovery, *mOS* median overall survival, *IDH* Isocitrate Dehydrogenase, *NR* not reached, *sAML* secondary acute myeloid leukemia.

## Targeting IDH2 in AML

Enasidenib (IDHIFA, formerly AG-221) is the first-in-class, selective and orally available mutant IDH2 inhibitor which demonstrated efficacy both in vitro and in vivo; normalizing 2-HG levels and inducing myeloid differentiation in various pre-clinical models^19^. In the first-in-human phase 1 dose escalation and expansion study (AG221-001), 345 patients with advanced *IDH2*-mutated myeloid malignancies were enrolled. The recommended phase 2 dose was determined as 100 mg daily, and in 214 patients with relapsed or refractory AML treated at the 100 mg dose level, the complete remission (CR) rate was 19.6% and CR with incomplete hematologic recovery (CRi) was 9%, with an overall response rate (ORR) of 39%^20^. The time to best response was 3.7 months, and median duration of CR/CRi lasted 8 months. The median OS among all patients was 8.8 months, with a median OS of 22.9 months for patients attaining a CR and 10.6 months for patients with a non-CR response^21^. *IDH2*-mutational clearance, as assessed by digital-droplet PCR with a detection level of 0.04%, occurred in ~1/3 of patients who attained aa CR or a CR with partial hematologic recovery (CRh) demonstrating the potential for deep remission in a minority of responding patients^21^.

Subsequent translational analysis of the original relapsed/refractory (R/R) cohort has identified that patients with co-occurring RAS pathway mutations (i.e., *NRAS, KRAS, FLT3-ITD, PTPN11*) are less likely to respond, as are patients with a high co-mutational burden (i.e. the presence of 6 or more co-occurring mutations)^22^. Patients with 3 or less mutations and no RAS pathway mutation had a 55% chance of responding to enasidenib monotherapy (with a 29% CR rate) whereas patients with ≥6 co-occurring mutations had an ORR of 31% (16% CR rate).

Of interest, while effective 2HG reduction was more frequent in IDH2-R140 variants (median 93% 2HG inhibition) than in IDH2-R172 (median 28% 2HG inhibition), equivalent responses occurred in both R140 and R172 patients^22^. Additionally and of some surprise, responses occurred independently of the size of the IDH2 clone at treatment initiation, as measured by variant allelic frequency at enrollment.

### Newly diagnosed *IDH2*-mutated AML

The original AG221-001 enasidenib monotherapy study enrolled 39 patients with newly diagnosed/treatment-naive *IDH2*-mutated AML, with a median age of 77 years and 23 (59%) of patients having an antecedent hematologic disorder such as MDS. In this subgroup, the CR rate was 18%, CR/CRi rate was 21%, ORR was 31%, and median OS was 11.3 months in this older and generally high-risk population^23^. Notably, response rates with single agent enasidenib were similar in previously untreated patients and those who progressed on prior therapy as discussed above. This could reflect the fact that previous standard, non-venetoclax-based therapies used in these studies do not alter the mechanisms of response or resistance to IDH inhibition.

Synergistic activity of enasidenib in combination with the hypomethylating agent azacytidine has been demonstrated pre-clinically, leading to the AG221-005 phase II randomized study of azacytidine + enasidenib, versus azacytidine alone, for newly diagnosed patients with AML ineligible for standard intensive chemotherapy. The primary study endpoint was overall response rate, with OS and EFS as key secondary endpoints. A total of 101 patients were enrolled in a 2:1 ratio to azacytidine + enasidenib (*n* = 68) or azacitidine (*n* = 33), with a CR rate of 53% vs 12%, CR/CRi rate of 63% vs 24%, and ORR of 71% vs 42% leading to the study reaching its primary endpoint. With a median follow up of 14 months, median EFS was 17.2 months with the combination vs 10.8 months with azacytidine alone (*p* = NS) and OS 22 months in both groups^24^.

When considering the overlapping survival of both frontline treatment arms, it is of particular importance to recall that the study was not blinded. The median number of cycles received of the enasidenib + azacytidine combination was 10, compared to 6 with azacytidine monotherapy, and at least 24% of patients randomized to azacytidine alone were taken off the study to receive subsequent enasidenib, alone or in combination, potentially confounding OS results.

For newly diagnosed younger patients appropriate for cytotoxic therapy, a recently reported Phase 1 combination study evaluated enasidenib in combination with 7 + 3 therapy^25^. In 91 patients with *IDH2* mutations and a median age of 63 yrs, CR/CRi rates were 74% and median OS was 25.6 months. A confirmatory Phase 3 study to clarify the role of adding enasidenib to induction/consolidation chemotherapy for *IDH2*-mutated AML is under evaluation, in a randomized phase 3 multi-institutional trial (NCT#03839771), which importantly will also include a maintenance component of enasidenib (vs placebo) after completion of induction/consolidation (+/− allogeneic SCT) to investigate the role of enasidenib as maintenance. A combination study of the liposomal 7 + 3 chemotherapeutic CPX-351 with enasidenib is also enrolling (NCT03825796).

### Targeting IDH1 in AML

Ivosidenib (TIBSOVO, formerly AG-120) is the first-in-class, selective and orally available mutant IDH1 inhibitor, with confirmed pre-clinical efficacy leading to robust 2HG inhibition and reinstatement of effective myeloid differentiation^26^. In the first-in-human phase 1 dose escalation and expansion study (AG120-001), 258 patients with advanced *IDH1*-mutated myeloid malignancies were enrolled^27^. The recommended phase 2 dose of ivosidenib was established at 500 mg daily, with a CR/CRh rate of 30% and ORR 42% in 125 R/R AML patients, with a median duration of CR/CRh lasting 8.2 months. The median OS among all patients was 8.8 months, with a median OS of 18 months in patients attaining a CR/CRh. IDH1-mutational clearance, as assessed by digital-droplet PCR with detection of 0.04%, occurred in 21% of responding patients, again highlighting the potential for deep remissions in a minority of patients. Patients with mutation clearance had longer durations of remission and longer overall survival with a median OS of 14.5 months vs 10.2 months in those without mutation clearance.

Similar to enasidenib, correlative translational analyses confirmed that patients with co-occurring RAS pathway mutations are less likely to respond to ivosidenib monotherapy. Interestingly, a favorable correlation was found between the presence of a J*AK2* mutation and response, with 7 of 11 (64%) AML with *JAK2*-V617F mutations achieving a CR or CRh^28^.

Clonal hierarchy was investigated by examining the baseline variant allele frequency (VAF) of the *IDH1* mutation in relationship to other identified gene mutations, with the *IDH1* mutation defined as sub-clonal in samples where a co-mutation was present at a VAF ≥ 5% that of the *IDH1* VAF, and otherwise the *IDH1* was defined as clonal. The *IDH1* mutation was determined to be sub-clonal in 28% of patients (and clonal in 72%), and with no association of clonal hierarchy to ivosidenib response was identified^28^.

### Newly diagnosed *IDH1*-mutated AML

There were 33 patients with newly diagnosed/treatment-naïve *IDH1*-mutated AML that were treated on the original AG120-001 ivosidenib monotherapy trial. The median age was 77 years and 26 (76%) had secondary AML, including 16 patients who had received prior hypomethylating agents for an antecedent hematologic disorder. In this high-risk group, the CR rate was 30%, CR/CRh was 42.5%, and with a median OS of 12.6 months^29^. In addition to the relapsed *IDH1*-mutated AML population, the United States Food and Drug Administration (FDA) has approved ivosidenib as monotherapy for the newly diagnosed older and chemotherapy-ineligible population based on these results.

Pre-clinical investigation of mutant *IDH1* transformed cell lines demonstrated enhanced differentiation and apoptosis with the combination of azacytidine and ivosidenib (Yen, Cancer Res 78;2018 suppl; abstr 4956). This led to a Phase 1b clinical trial which enrolled 23 patients to the combination of azacytidine and ivosidenib, demonstrating a CR rate of 61%, CR/CRh rate of 70%, and a 12-month OS of 82% (median duration of response or DOR has not been reached, with median follow-up of 16 months)^30^. The follow-up confirmatory Phase 3 AGILE study of ivosidenib vs placebo in combination with azacytidine in untreated AML is now ongoing (NCT 03173248) with a primary endpoint of relapse-free survival. Ultimately, data from this combination should be compared to venetoclax regimens, the current standard of care for this population. Conceptually, the combination of a hypomethylating agent with venetoclax and an IDH inhibitor should offer the highest chance of response, decrease chance of resistance and provide long-term remissions. We believe that an effective combination used early in the disease process would be superior to a sequential approach of these effective agents as we’ve learned from acute lymphoblastic leukemia or multiple myeloma.

The Phase 1 combination study of intensive chemotherapy + enasidenib for newly diagnosed patients discussed above also included an arm of *IDH1*-mutated AML, that received ivosidenib in combination with standard intensive chemotherapy. In 60 patients with *IDH1* mutations and a median age of 63 yrs, CR/CRi rates were 77%, and 12-month OS was 78%^25^. A confirmatory randomized phase 3 trial (NCT#03839771) is under evaluation, which also includes maintenance ivosidenib (vs placebo) to evaluate the utility of ivosidenib as maintenance after consolidation therapy (+/− allogeneic stem cell transplant). A study of CPX-351 in combination with ivosidenib (NCT04493164) is also currently enrolling.

### Second generation IDH inhibitors

Additional small molecule IDH inhibitors are under various stages of preclinical and clinical development. Compounds from Novartis (IDH305), Agios (AG881; now vorasidenib) and Bayer (BAY1436032) were previously evaluated in Phase 1 clinical trials, and are not currently under clinical development in hematologic malignancies^31,32^. The brain-penetrant dual inhibitor of IDH1 and IDH2, vorasidenib, is currently under development in recurrent/refractory *IDH*-mutated glioma.

Olutasidenib, formerly FT-2102, warrants specific mention as a well-tolerated and effective mutant IDH1 inhibitor^33^. At the recommended dose of 150 mg twice daily in R/R AML, an ORR of 41% (18% CR) as monotherapy, and ORR of 46% (12% CR) in combination with azacytidine was reported. (*Watts ASH 2019*) A recent press release in October 2020 of top-line data from a planned interim analysis of the pivotal Phase 2 olutasidenib monotherapy arm for R/R AML stated positive results; in 123 R/R AML patients, a CR/CRh rate of 33.3% (30% CR and 3% CRh) and an impressive DOR (censored at HSCT) of 13.8 months was attained. This data has not yet been presented.

### Adverse events

In general, the IDH inhibitors are well tolerated oral therapies. Enasidenib is associated with an indirect hyperbilirubinemia which occurs in approximately 15–20% (grade ≥ 3 in 8%), related to off-target UGT1A1 inhibition^20^. This is clinically similar to Gilbert’s syndrome and is generally not clinically significant. Ivosidenib is associated with grade ≥3 QTc prolongation in 7–8%, and routine EKG assessment is recommended along with discontinuation of other offending QTc prolonging concomitant medications when possible^27^.

Both IDH inhibitors are associated with development of differentiation syndrome (DS), which is reported in approximately 12–15% of patients receiving an IDH inhibitor as monotherapy and is most frequently manifested as dyspnea, culture-negative fevers, pulmonary infiltrates, and/or hypoxia^34^. IDH-inhibitor related DS occurs at a median of 30 days of treatment, however DS can be delayed and can occur several months into therapy, so attention is warranted. In a review by the FDA of the pivotal trials of ivosidenib and enasidenib, DS was identified in 19% of patients receiving either of those agents^35^. Discontinuation of the IDH inhibitor in the setting of a severe IDH-DS is often recommended, however the half-life of the IDH inhibitors is measured in days (not hours) and thus the primary treatment of IDH-DS should be with corticosteroids (dexamethasone 10 mg BID typically recommended) with supportive care as also required. Several reviews of DS have been published^34,36,37^.

### Resistance mechanisms

As mentioned previously, co-occurring mutations in the RAS pathway are associated with both primary and secondary therapeutic resistance, to both enasidenib and ivosidenib^22,28^.

Mutations in transcription factors involved in differentiation (i.e., *RUNX1, CEBPA, GATA2*) and/or chromatin regulators (i.e., *DNMT3A, ASXL1*) are also seen in up to 1/3 of patients with acquired relapse.

Similar to the T315I mutation occurring in the setting of tyrosine kinase inhibitors targeting Bcr-Abl, or the F691L gatekeeper mutation associated with FLT3 inhibitor resistance, emergence of second-site mutations affecting the binding site of IDH2 (i.e., Q316E and I319M) and IDH1 (i.e. D279N, S280F) have also been described in responding patients in the setting of therapeutic resistance^28,38^.

Finally, acquired resistance through a resistance pathway termed “isoform switching” has also been described, wherein an *IDH1* mutation is detected at the time of relapse in a patient with an *IDH2* mutation treated with a targeted IDH2 inhibitor, and vice-versa^39,40^. Both second-site mutations and isoform switching are associated with a rising 2HG level at relapse.

Single cell DNA sequencing analyses have demonstrated that relapse frequently occurs due to multiple different resistance mechanisms occurring in parallel. Ultimately, these patterns of IDH inhibitor resistance support the ongoing evaluation and use of combination therapies to prevent resistance to IDH inhibitor monotherapy.

### Venetoclax for patients with IDH mutations

The BCL2 inhibitor venetoclax, in combination with hypomethylating agent therapy has quickly become a standard of care treatment option for newly diagnosed, older and intensive chemotherapy-ineligible patients with AML, due to clinically meaningful improvements in remission rates and overall survival^41^. From the beginning of venetoclax development in AML, it has been apparent that patients with *IDH1* and *IDH2* mutations respond particularly well to venetoclax-based therapies^9^. Updated results of *IDH1* and *IDH2* mutated patients to azacytidine + venetoclax was reported at the Annual Society of Hematology Meeting in 2020 and confirm these impressive clinical results, with *IDH1* mutated patients experiencing a CR/CRh of 59.4% and median DOR of 29.6 months, with a median OS of 17.5 months. Patients with *IDH2* mutations experienced a CR/CRh of 79.6% with both a median DOR and median OS that has not yet been reached, with a 12-month OS of 75%^42^.

As both IDH inhibitors and venetoclax are effective in patients with *IDH* mutations, there is increasing interest in the sequencing or combination of these effective and potentially complementary therapies. Given the exquisite sensitivity of *IDH*-mutated AML to venetoclax combinations, and the ability to target other cooperating mutations or clones susceptible to BCL2 inhibition in this AML subtype, we favor using the combination of a hypomethylating agent with venetoclax in patients above the age of 65, able to tolerate this therapy. Conversely, we recommend reserving use of single agent IDH inhibitors in older patients (age > 75 years) or those with significant comorbidities (Fig. 2). A clinical trial of ivosidenib + venetoclax ± azacytidine is ongoing; interim trial results were presented at American Society of Clinical Oncology meeting in 2020 and demonstrated a composite remission rate of 80% in patients with both newly diagnosed and relapsed *IDH1*-mutated myeloid malignancies, with 50% of patients attaining minimal or measurable residual disease (MRD) negativity^43^. Updated trial results with longer follow-up are anticipated in the coming year. In addition, a trial of enasidenib in combination with venetoclax (NCT04092179) is now enrolling.

## Conclusion

In conclusion, the development of oral, potent, small molecule and mutant specific IDH1 and IDH2 inhibitors represents an early success story of the cancer genome-sequencing era, and signifies an important advance for AML therapy in the era of personalized therapeutics. Evaluation of strategies to increase efficacy and prevent relapse with IDH inhibitors are ongoing, including clinical investigations of treatment combinations with standard anti-leukemia therapies (i.e., intensive chemotherapy, hypomethylating agents) as well as rational combinations (venetoclax) and agents targeted against AML resistance pathways (i.e., FLT3, RAS, other RTK pathway inhibitors). In addition, second generation and “pan” IDH1/IDH2 inhibitors are also under development, providing further hope in the promise of increasingly improved outcomes in patients with *IDH*-mutated AML.
